# Elucidating the Molecular Mechanisms of Pulsed Light-Induced Lycopene Accumulation in Tomatoes Through Integrated Multi-Omics Analysis

**DOI:** 10.3390/ijms262411828

**Published:** 2025-12-07

**Authors:** Guangning Na, Yeting Sun, Xueshan Wen, Chao Zhang, Xiaoyan Zhao

**Affiliations:** 1 College of Food Science, Shenyang Agricultural University, Shenyang 110866, China; naguangning@situ.edu.cn (G.N.);; 2 Institute of Agri-Food Processing and Nutrition, Beijing Academy of Agriculture and Forestry Sciences, Beijing Key Laboratory of Fruits and Vegetables Preservation and Processing, Key Laboratory of Vegetable Postharvest Processing, Ministry of Agriculture and Rural Affairs, Beijing 100097, China

**Keywords:** *Solanum lycopersicum* L., lycopene, pulsed light, transcriptomics, proteomics, metabolomics

## Abstract

Tomato (*Solanum lycopersicum* L.) is rich in the antioxidant lycopene, which often degrades postharvest. Pulsed light shows promise in preserving lycopene, yet its molecular mechanisms remain unclear. This study integrates transcriptomics, proteomics, and metabolomics to elucidate how pulsed light affects lycopene synthesis in tomatoes. The results showed that lycopene content increased significantly in pulsed light-treated tomatoes. Transcriptomic analysis identified 1092 significantly differentially expressed genes (DEGs), proteomic analysis identified 1046 significantly differentially accumulated proteins (DAPs), and metabolomic analysis identified 272 significantly differentially accumulated metabolites (DEMs). These were significantly enriched in pathways such as terpenoid backbone biosynthesis, carotenoid biosynthesis, the tricarboxylic acid cycle (TCA), and photosynthesis. The upregulation of eight key genes central to lycopene biosynthesis was validated by qRT-PCR, confirming their involvement in the observed accumulation. Integrated multi-omics analysis revealed coordinated regulation of photosynthesis, carbohydrate metabolism, and terpenoid synthesis, highlighting the reprogramming of energy metabolism and secondary metabolite synthesis in lycopene accumulation. This study provides a comprehensive understanding of the molecular mechanisms by which pulsed light enhances lycopene content in tomatoes. The findings suggest that pulsed light treatment activates key metabolic pathways, leading to increased lycopene synthesis. This research offers a theoretical basis for optimizing pulsed light technology and developing new preservation strategies to maintain and enhance the nutritional quality of tomatoes during postharvest storage.

## 1. Introduction

Tomato (*Solanum lycopersicum* L.), as a globally cultivated and consumed horticultural crop, is not only an essential component of daily diets but also attracts significant attention due to its rich nutritional content [1]. Lycopene, the primary carotenoid in tomatoes, imparts the characteristic red color to the fruit. It possesses excellent antioxidant capacity, effectively scavenging free radicals in the body, slowing cellular aging, reducing the risk of chronic diseases, and potentially positively impacting cardiovascular health and cancer prevention [2]. Additionally, tomatoes are rich in ascorbic acid, vitamin E, potassium, and various phenolic compounds. These nutrients work synergistically, making tomatoes an indispensable food for promoting human health [3].

During postharvest storage, lycopene content in tomatoes often gradually decreases due to environmental factors, microbial activity, and the fruit’s physiological metabolism, leading to a decline in nutritional value and quality, and causing economic losses to farmers and related industries [4]. Therefore, exploring effective preservation techniques to reduce lycopene loss is of significant economic and practical importance. In recent years, pulsed light, as an emerging non-thermal treatment technology, has gradually gained attention in food preservation research [5]. Pulsed light, through photochemical and photothermal effects generated by high-intensity short pulses, can not only effectively kill microorganisms and extend the shelf life of tomatoes but may also influence lycopene synthesis and stability [6]. Some studies have shown that pulsed light treatment can maintain or even increase lycopene content to a certain extent [7,8]. However, the mechanisms by which pulsed light affects lycopene are not yet fully understood. In-depth research will help optimize pulsed light technology parameters and provide theoretical support for its widespread application in tomato preservation.

With the continuous development of omics technologies, multi-omics integrated analysis provides a powerful tool for deeply exploring the molecular mechanisms of lycopene synthesis and accumulation [9]. Proteomics can identify and quantify key enzymes and regulatory proteins involved in lycopene synthesis, revealing the fruit-ripening mechanism [10]. Transcriptomics can analyze differences in gene expression in tomato, uncover potential regulatory factors, and identify changes in signaling pathways [11]. Metabolomics can comprehensively monitor the dynamic changes in lycopene, its precursors, and related metabolites, thereby constructing a metabolic network of how pulsed light affects lycopene synthesis [12]. By integrating multi-omics data, a comprehensive analysis from gene expression, protein synthesis, to metabolite production can be achieved, providing a systematic and in-depth perspective for revealing the mechanisms of pulsed light action.

This study focuses on the impact of pulsed light on lycopene synthesis mechanisms, employing a combined transcriptomics, proteomics, and metabolomics approach to comprehensively analyze physiological and biochemical changes in tomatoes following pulsed light treatment. The aim is to reveal how pulsed light regulates gene expression, protein function, and metabolic pathways to influence lycopene synthesis and accumulation, providing a key theoretical basis for tomato storage and preservation. The research results will help develop tomato preservation strategies based on pulsed light technology, reduce lycopene loss during storage, enhance the nutritional value and quality of tomatoes, promote the high-quality development of the tomato industry, and provide useful references for research on the preservation of other fruits and vegetables.

## 2. Results

### 2.1. Determination of Lycopene Changes in Tomatoes Under Different Treatments

**Figure 1:**
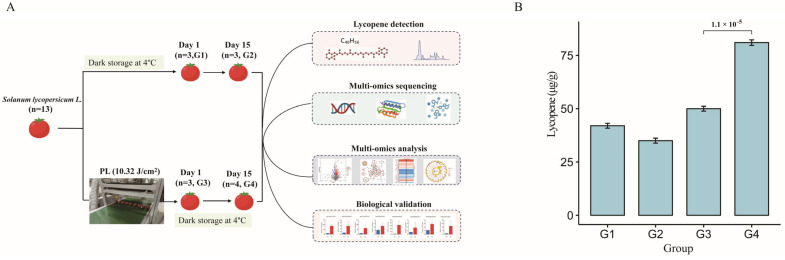
(**A**) The changes in the appearance of tomatoes and lycopene content under different treatments. (**B**) Lycopene content in tomatoes under four different groups. Statistical significance was determined by Student’s *t*-test (*p* < 0.05).

The detailed workflow of the entire study is presented in Figure 1A. To quantify these changes and assess the underlying biochemical alterations, this study employed UPLC-MS/MS to measure lycopene content in tomato samples from the four groups. The results showed that the initial lycopene content (group G1) was 42 mg/kg. After 15 days of storage under dark conditions without any treatment (group G2), the lycopene content decreased to 35 mg/kg, showing a declining trend, though it was not statistically significant (*p* > 0.05). In contrast, samples treated with pulsed light for 1 h (group G3) exhibited a significant increase in lycopene content, reaching 50 mg/kg. Notably, samples treated with pulsed light followed by 15 days of storage (group G4) showed a further rise in lycopene content to 81 mg/kg, with a statistically significant difference compared to group G3 (*p* < 0.05). These results conclusively demonstrate that pulsed light treatment significantly enhances lycopene levels in tomatoes during postharvest storage.

### 2.2. Metabolite Identification and Functional Annotation

**Figure 2:**
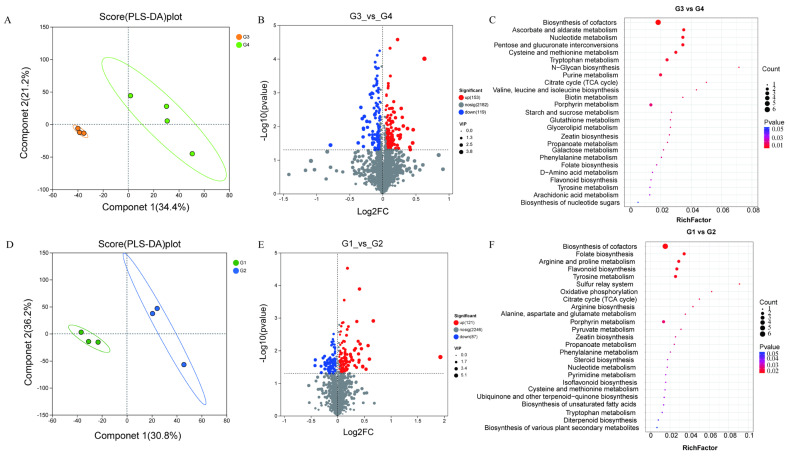
Metabolomics analysis. (**A**) PLS-DA analysis of G3 and G4 groups; (**B**) volcano plot of differential metabolites between G3 and G4 groups; (**C**) KEGG enrichment analysis of significantly different metabolites between G3 and G4 groups. (**D**) PLS-DA analysis of G1 and G2 groups; (**E**) volcano plot of differential metabolites between G1 and G2 groups; (**F**) KEGG enrichment analysis of significantly different metabolites between G1 and G2 groups.

To gain a deeper understanding of the impact of pulsed light on lycopene, a comprehensive untargeted metabolite analysis was conducted using ultra-high-performance liquid chromatography (UHPLC). Initially, we analyzed the metabolic changes between groups G3 and G4. The comparison was performed using partial least squares discriminant analysis (PLS-DA) to elucidate the specific differences between the groups. The results showed that the principal components generated by the PLS-DA analysis accounted for approximately 34.4% and 21.2% of the variance (Figure 2A). The PLS-DA analysis based on metabolites revealed significant differences between the two groups after pulsed light treatment and storage. During DAM screening, we identified 272 significantly altered metabolites, including 153 upregulated and 119 downregulated metabolites (Figure 2B). Preliminary analysis indicated that the DAMs included porphyrins, tricarboxylic acids and their derivatives, sesquiterpenoids, carbohydrates and carbohydrate conjugates, pyrimidine nucleotide sugars, and carbonyl compounds, with a clear upregulation trend observed after pulsed light treatment and storage. KEGG pathway enrichment analysis revealed that the significantly upregulated metabolites in group G4 were mainly enriched in porphyrin metabolism, cysteine and methionine metabolism, citrate cycle (TCA cycle), and biosynthesis of cofactors (Figure 2C). Additionally, we performed metabolomic analysis on groups G1 and G2. PLS-DA demonstrated a clear distinction between the two groups (Figure 2D). There were 209 significantly different metabolites between the two groups (Figure 2E). Notably, the KEGG enrichment analysis of the differential metabolites between G1 and G2 revealed that the significantly downregulated metabolites in group G2 were enriched in flavonoid biosynthesis, citrate cycle (TCA cycle), ubiquinone and other terpenoid-quinone biosynthesis, and oxidative phosphorylation (Figure 2F). This indicates that these pathways play a crucial role in lycopene synthesis.

### 2.3. Proteomic Analysis

**Figure 3:**
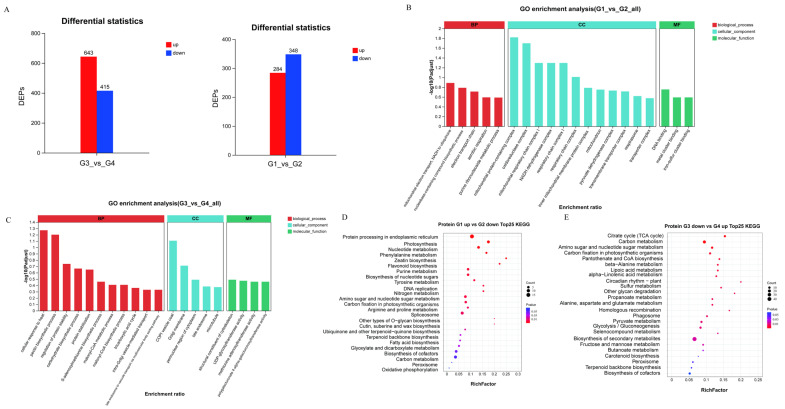
Proteomics analysis. (**A**) Bar chart of significantly different proteins between G1 and G2, and G3 and G4 groups; (**B**) GO analysis of differential proteins between G1 and G2 groups; (**C**) GO analysis of differential proteins between G3 and G4 groups; (**D**) KEGG analysis of significantly downregulated differential proteins in G2 compared to G1; (**E**) KEGG analysis of upregulated differential proteins in G4 compared to G3.

To further explore the molecular mechanisms underlying the effect of pulsed light on lycopene synthesis in tomatoes, we conducted proteomic analysis on the same four groups as in the metabolomic analysis, identifying proteins based on differences in their accumulation levels. The results showed that there were 632 significant DAPs between groups G1 and G2 (284 upregulated and 348 downregulated), and 1058 DAPs between groups G3 and G4 (643 upregulated and 415 downregulated) (Figure 3A). GO and KEGG analyses revealed that the GO enrichment terms for the differential proteins between G1 and G2 included mitochondrial electron transport, NADH to ubiquinone, oxidoreductase complex, and NADH dehydrogenase complex (Figure 3B). In contrast, the differentially expressed proteins between G3 and G4 were significantly enriched in the tricarboxylic acid cycle, carbohydrate biosynthetic process, and malonyl-CoA metabolic process (Figure 3C). KEGG enrichment results showed that, compared to group G3, the down-accumulated proteins in group G4 were mainly enriched in the citrate cycle (TCA cycle), carbon metabolism, carbon fixation in photosynthetic organisms, pantothenate and CoA biosynthesis, biosynthesis of secondary metabolites, butanoate metabolism, carotenoid biosynthesis, peroxisome, terpenoid backbone biosynthesis, and biosynthesis of cofactors (Figure 3D). The downregulated proteins in group G2 were mainly enriched in photosynthesis-related pathways, particularly in the core processes of carbon fixation and terpenoid backbone biosynthesis, as well as in the biosynthesis of cofactors and carbon metabolism (Figure 3E). These results indicate that the loss of lycopene during long-term storage of tomatoes may be associated with the inhibition of pathways such as photosynthesis and carbon metabolism, whereas the increase in lycopene after pulsed light treatment is associated with the activation of energy metabolism and terpenoid biosynthesis. These results indicate that the loss of lycopene during long-term storage of tomatoes may be related to the inhibition of photosynthesis-related proteins (particularly photosystem I/II and photosynthesis-antenna proteins) and key carbon metabolism processes (including the TCA cycle and Calvin cycle), leading to insufficient supply of energy (ATP) and reducing power (NADPH). In contrast, the increase in lycopene after pulsed light treatment is associated with the activation of energy metabolism and terpenoid biosynthesis.

### 2.4. Transcriptomics and Functional Annotation of DEGs

**Figure 4:**
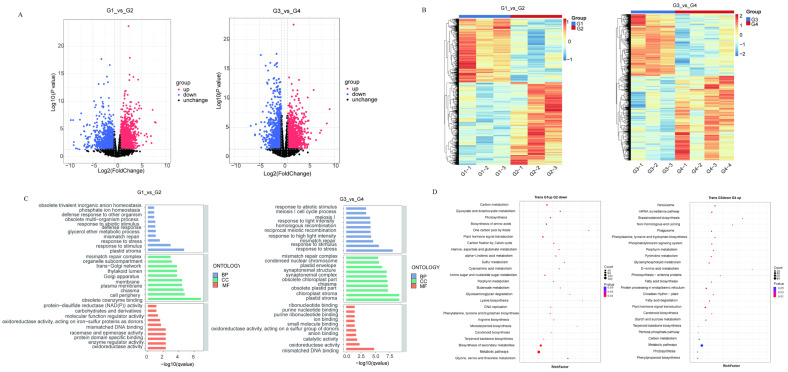
Transcriptome analysis. (**A**) Volcano plot of differentially expressed genes between G1 and G2, and between G3 and G4. (**B**) Heatmap of hierarchical clustering of differentially expressed genes between G1 and G2, and between G3 and G4; (**C**) GO enrichment analysis of differentially expressed genes between G1 and G2, and between G3 and G4; (**D**) KEGG enrichment analysis of differentially expressed genes between G1 and G2, and between G3 and G4.

To identify lycopene-related genes and explore the molecular mechanisms by which pulsed light affects lycopene synthesis, we conducted RNA-seq transcriptomic analysis of four groups of tomato samples. Differential expression gene analysis revealed that there were 3262 DEGs in groups G3 and G4 (2102 upregulated and 1160 downregulated), while groups G1 and G2 had only 1683 DEGs (1003 upregulated and 860 downregulated) (Figure 4A). The significantly fewer DEGs between G1 and G2 than between G3 and G4 suggest that lycopene metabolism is more sensitive to pulsed light treatment. A heatmap showing genes differentially expressed between the two groups is presented (Figure 4B).

We performed GO analysis of the DEGs across three major categories: cellular component (CC), biological process (BP), and molecular function (MF). Figure 4C, presented in a circular diagram format, shows that in the G3 vs. G4 group, 10 GO terms were significantly enriched in the BP category, including response to high light intensity, response to light intensity, response to stress, and response to stimulus. Similarly, the top 10 significantly enriched GO terms in the MF category were selected, with oxidoreductase activity, acting on a sulfur group of donors, disulfide as acceptor, and mismatched DNA binding showing the highest enrichment significance. Additionally, plastid stroma was the most significantly enriched category in CC. In the G1 vs. G2 group, GO terms such as response to stress, response to stimulus, enzyme regulator activity, oxidoreductase activity, acting on a sulfur group of donors, and disulfide as acceptor, were significantly enriched.

To further analyze the functions of the DEGs, we conducted KEGG enrichment analysis (Figure 4D). The results showed that, compared to G3, genes significantly upregulated in G4 were mainly enriched in carotenoid biosynthesis, photosynthesis antenna proteins, and terpenoids, etc. Compared to G1, genes significantly downregulated in G2 were mainly enriched in carbon metabolism, carbon fixation by Calvin cycle, and monoterpenoid biosynthesis.

### 2.5. Integrated Proteomic and Transcriptomic Analysis

**Figure 5:**
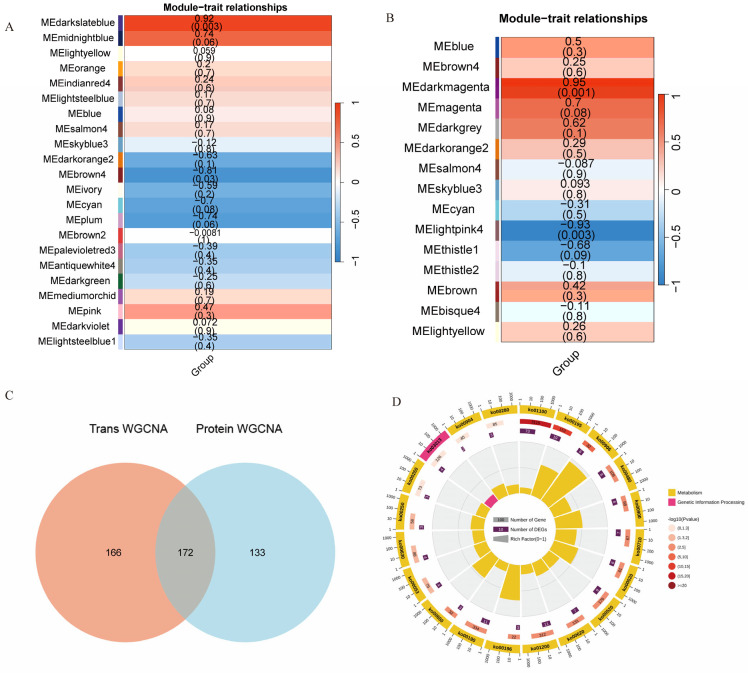
Integrated transcriptomics and proteomics analysis. (**A**) WGCNA analysis of differential proteins between G3 and G4 groups; (**B**) WGCNA analysis of differential genes between G3 and G4 groups; (**C**) Venn diagram analysis of genes significantly related to lycopene at both the transcriptomic and proteomic levels; (**D**) KEGG enrichment analysis of common genes between proteomic and transcriptomic levels related to lycopene.

To further investigate the potential molecular mechanisms underlying lycopene synthesis regulated by pulsed light and to reveal the synergistic relationships at the transcriptional and translational levels, we performed a joint analysis of proteomic and transcriptomic data from groups G3 and G4. Proteomic analysis using WGCNA identified 22 distinct gene modules, each with a unique gene expression pattern (Figure 5A). Correlation analysis between each module and lycopene expression revealed that the MEdarkslateblue module was highly positively correlated with lycopene expression levels (correlation = 0.92, *p* < 0.05). RNA-seq data were processed using WGCNA, and coexpressed gene modules were identified. The results showed that among the 15 modules identified, the MEdarkmagenta module was highly positively correlated with lycopene expression levels (correlation = 0.95, *p* < 0.05) (Figure 5B). By the intersection of the gene sets from the significantly correlated modules identified in the proteomic and transcriptomic analyses, 172 common genes were identified (Figure 5C). KEGG analysis of the common genes showed that they were mainly enriched in a coordinated metabolic network supporting lycopene synthesis. This network includes the upstream terpenoid backbone biosynthesis pathway that supplies fundamental precursors, the downstream carotenoid biosynthesis pathway responsible for lycopene formation, and essential supporting processes including oxidative phosphorylation, photosynthesis antenna proteins, carbon fixation by the Calvin cycle, and multiple primary metabolic pathways (Figure 5D).

### 2.6. Integrated Metabolomic and Transcriptomic Analysis

**Figure 6:**
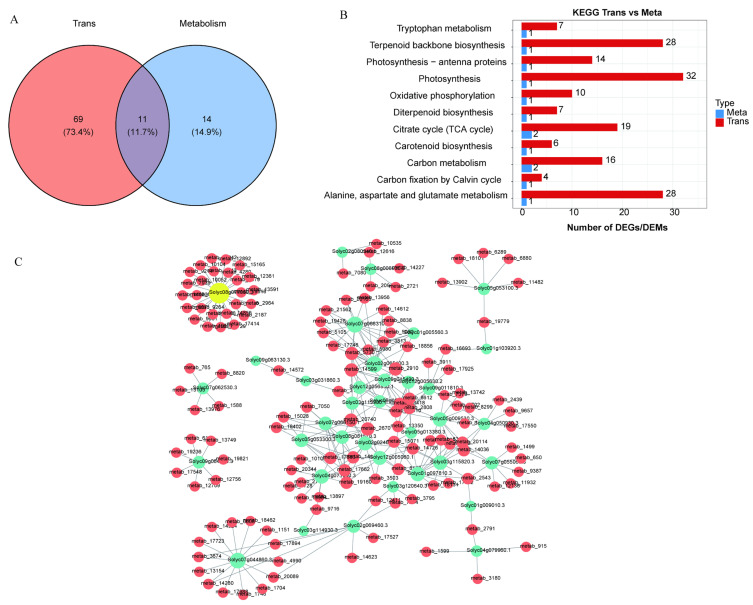
Integrated transcriptomics and metabolomics analysis. (**A**) Venn diagram of shared and unique KEGG pathways between metabolomics and transcriptomics. (**B**) Analysis of 11 shared KEGG pathways enriched in both metabolomics and transcriptomics. (**C**) Correlation network map of lycopene-related genes and metabolites. Red nodes represent metabolites, and green nodes represent genes.

We performed a combined transcriptomic and metabolomic analysis to better understand the molecular mechanisms by which pulsed light affects lycopene synthesis. Firstly, we took the intersection of the pathways enriched by DEGs and DAMs between groups G3 and G4, resulting in 11 shared metabolic pathways, including arachidonic acid metabolism, ascorbate and aldarate metabolism, biotin metabolism, cysteine and methionine metabolism, folate biosynthesis, galactose metabolism, and glutathione metabolism (Figure 6A,B). To further elucidate the molecular mechanisms underlying the effects of pulsed light on lycopene synthesis, we performed a comprehensive correlation analysis of key pathway genes and metabolites (Figure 6C). This analysis revealed several significant associations between metabolites and differentially expressed genes involved in photosynthesis, terpenoid backbone biosynthesis, and carotenoid biosynthesis. Specifically, the metabolites significantly correlated with genes involved in photosynthesis included *Solyc06g054260.1* with Phosphoenolpyruvic Acid, *Solyc02g069460*.3 with Glycerophosphocholine, and *Solyc08g006930*.3 with 4-Hydroxybenzoic Acid. In the terpenoid backbone biosynthesis pathway, the most significant correlations were observed between *Solyc08g081570*.3 and 5-Aminolevulinic acid, *Solyc04g079960*.1 and D-Ribulose 5-phosphate, and Solyc03g115980.1 and Sitosterol 3-O-acyl-glucoside. For carotenoid biosynthesis, the most significant metabolite-gene correlations were identified between *Solyc04g050930*.3 and Sclareol oxide, and *Solyc04g071940*.3 and 4-oxo-Retinoic acid. These findings highlight the interplay between specific genes and metabolites in these pathways, suggesting their potential roles in modulating lycopene synthesis following pulsed light treatment.

### 2.7. Integrated Multi-Omics Analysis

**Figure 7:**
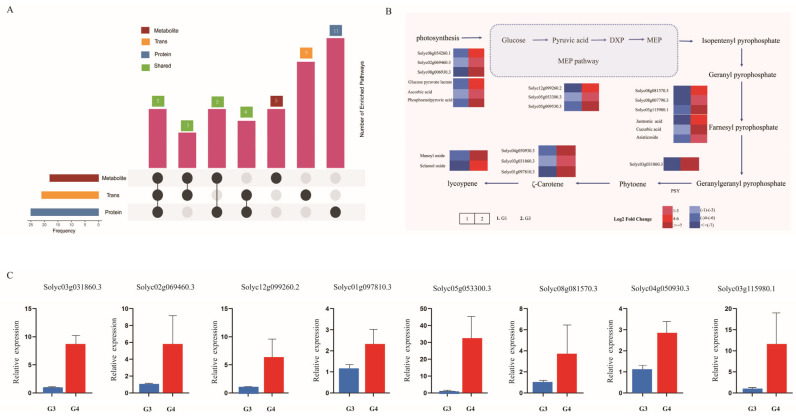
Statistical and functional integration of transcriptome, proteome, and metabolome data. (**A**) The differentially expressed genes, proteins, and metabolites between G3 and G4 were significantly enriched in various KEGG pathways. The pink bars represent the number of metabolic pathways shared by each omics layer. (**B**) The lycopene biosynthesis pathway under different treatments in G3 and G4 groups. Differentially synthesized genes, proteins, and/or metabolites between the two groups are shown. The color scale for different levels of differential expression is given at the bottom. (**C**) qRT-PCR analysis of key genes.

In this study, we integrated multi-omics data through a comprehensive analysis of transcriptomic, proteomic, and metabolomic profiles. Focusing on the DEGs, DEPs, and DEMs between groups G3 and G4, the study identified 1092 DEGs, 1046 DEPs, and 82 DEMs, which were significantly enriched in 42 KEGG pathways (Figure 7A). The analysis revealed synergistic regulatory patterns among genes and proteins, genes and metabolites, or proteins and metabolites in photosynthesis, carbohydrate metabolism, terpenoid backbone biosynthesis, energy metabolism, and carbon metabolism. Among these, the citrate cycle (TCA cycle), terpenoid backbone biosynthesis, and carotenoid biosynthesis exhibited significant differential regulation between groups G3 and G4 (Figure 7B). Additionally, we experimentally validated the expression of key genes involved in lycopene biosynthesis, including *Solyc12g099260.2*, *Solyc01g097810*.3, *Solyc05g053300*.3, *Solyc04g050930*.3, *Solyc08g081570*.3, *Solyc03g031860*.3, *Solyc02g069460*.3, and *Solyc03g115980.1*. As shown in Figure 7C, the expression levels of these genes were significantly higher in group G4 compared to group G3, a trend that was highly consistent with our RNA-seq data (Supplementary Table S1), thus robustly cross-validating the reliability of the transcriptomic results.

## 3. Discussion

Lycopene is a key indicator of the color and nutritional value of ripe tomatoes, with strong antioxidant, anti-inflammatory, and cardiovascular protective functions [13,14]. However, it is highly susceptible to degradation during postharvest storage, leading to a decline in quality. Light pulse treatment has been shown to significantly increase lycopene content after storage, but the underlying molecular mechanisms remain unclear. In this study, using tomato fruit as the material, we integrated multi-omics technologies of transcriptomics, metabolomics, and proteomics to systematically elucidate the key pathways and regulatory networks induced by pulsed light for lycopene accumulation. A total of 127 differentially expressed genes, 1046 differentially abundant proteins, and 272 differential metabolites were identified, which were significantly enriched in pathways such as terpenoid backbone biosynthesis, carotenoid biosynthesis, TCA cycle, and cofactor biosynthesis. The upregulation of key genes was identified as the primary driver of lycopene accumulation. This study provides a theoretical basis and technical pathway for maintaining and enhancing the quality of postharvest tomatoes.

Plant metabolomics has been widely used to elucidate the remodeling patterns of metabolites across different treatments or storage stages [15,16] In this study, employing non-targeted metabolomics via UPLC-MS, 272 DAMs were identified between G3 and G4, with key differences covering porphyrins, TCA cycle derivatives, sesquiterpenoids, and carbohydrate synthesis. These results suggest that pulsed light induces a systematic reprogramming of energy and redox states, thereby promoting lycopene accumulation [17,18]. Further KEGG enrichment analysis revealed that the significantly upregulated DAMs in G4 were mainly enriched in porphyrin metabolism, cysteine-methionine metabolism, the TCA cycle, and cofactor biosynthesis. Porphyrin metabolism provides precursors for tetrapyrrole synthesis, which can directly affect the reducing power (NADPH) and energy (ATP) required for carotenoid synthesis [19]. Enhancing the TCA cycle and cofactor biosynthesis increases the supply of GGPP and the activity of phytoene synthase (PSY), thereby synergistically amplifying lycopene flux [20]. Conversely, in G2, downregulated metabolites were significantly enriched in flavonoid biosynthesis, the TCA cycle, ubiquinone and other terpenoid-quinone biosynthesis, and oxidative phosphorylation. Downregulation of these pathways may reduce carbon flux through IPP/DMAPP and GGPP, thereby reducing lycopene synthesis and increasing its degradation.

Additionally, transcriptomic and proteomics analyses revealed that, in tomato samples treated with pulsed light, pathways such as photosynthesis, carotenoid biosynthesis, terpenoid backbone biosynthesis, TCA cycle, and carbon metabolism were significantly enriched. Terpenoid biosynthesis, which is upstream of lycopene synthesis, may promote lycopene accumulation by increasing the supply of precursors for carotenoid synthesis, such as isoprenoid diphosphate (IPP) and dimethylallyl diphosphate (DMAPP) [21]. Studies have shown that terpenoid backbone biosynthesis is a core precursor source for carotenoid synthesis. Its key products, IPP and DMAPP, are direct precursors of lycopene synthesis, and activating terpenoid biosynthesis can significantly enhance lycopene synthesis efficiency [22,23]. Cofactors play a crucial role in lycopene synthesis. By regulating the supply and activity of these cofactors, lycopene yield can be significantly increased. In metabolic engineering and microbial synthesis, optimizing metabolic pathways by enhancing the supply of NADPH and acetyl-CoA can improve the efficiency of lycopene synthesis [24]. The regulatory role of photosynthesis in lycopene synthesis has been widely studied [25]. Moderate light exposure is beneficial for the synthesis of carotenoids, including lycopene, in plant tissues. Studies have shown that light can induce the formation of photosynthetic complexes, thereby indirectly promoting lycopene synthesis.

The integrated multi-omics analysis in this study provides new insights into light-regulated lycopene biosynthesis. Compared with previous studies [26], pulsed light exhibits unique regulatory characteristics, activating not only the carotenoid biosynthesis pathway but also synchronously enhancing the systemic coupling of the TCA cycle and antioxidant metabolism. Studies have shown that different light qualities influence carotenoid accumulation through specific photoreceptor signaling pathways [27]. In contrast, our findings reveal that pulsed light simultaneously activates both photosynthesis antenna proteins and terpenoid backbone synthesis, suggesting its broader photoreceptor activation capability. These findings indicate that distinct light-regulation methods may achieve precise modulation of metabolic pathways via distinct signal transduction pathways. Additionally, previous studies have shown that during lycopene synthesis, phytoene produced by PSY is converted into lycopene via a series of desaturation reactions catalyzed by enzymes such as phytoene desaturase and ζ-carotene desaturase [28]. Experimental validation revealed that the *PSY* gene was significantly upregulated 15 days after pulsed light treatment. Furthermore, through integrated analysis, metabolites Ascorbic acid, Manoyl oxide, Cucurbic acid, and Asiaticoside were identified as significantly correlated with lycopene synthesis, and changes in these metabolites were significantly associated with increased lycopene content after pulsed light treatment.

It is noteworthy that the metabolic reprogramming identified in this study may be triggered by early signaling events induced by pulsed light. We hypothesize that pulsed light initiates lycopene biosynthesis through the following mechanisms: on one hand, high-intensity pulsed light may transiently elevate intracellular reactive oxygen species (ROS) levels, and ROS as key signaling molecules could activate both the antioxidant defense system and secondary metabolic pathways by regulating transcription factors, ultimately promoting lycopene synthesis [29,30]. This hypothesis is supported by the significant activation of the ascorbic acid metabolism pathway observed in our study. On the other hand, the red/far-red wavelengths within the broad spectrum of pulsed light might be perceived by the phytochrome system, which could regulate the expression of key genes such as PSY through phytochrome [31,32]. Collectively, our findings provide a solid foundation for the future development of pulsed light applications in postharvest preservation. Based on the molecular mechanisms revealed in this study, future work will focus on exploring and optimizing novel pulsed light treatment strategies to more effectively enhance lycopene content.

## 4. Materials and Methods

### 4.1. Tomatoes Materials and Pulsed Light Treatments

The ‘Jinfan 3166’ tomatoes (*Solanum lycopersicum* L.) were provided by the Analysis and Testing Center of Xinjiang Academy of Agricultural and Reclamation Sciences. The tomatoes were harvested at the commercial red-ripe stage and transported under low-temperature conditions (4 °C) to the laboratory within 2 h postharvest. Thirteen tomatoes of uniform size, weight (average 75–85 g), and appearance, and without any physical damage or disease, were selected for the experiment. The tomatoes were immersed in a 100 ppm free chlorine solution for 2 min, then thoroughly rinsed with tap water and gently dried to remove surface moisture. Thirteen tomatoes uniform in size, weight, and appearance, and without any damage, were selected and temporarily stored at 4 ± 1 °C. For the pulsed light group, seven tomatoes were treated with pulsed light using the Steribeam Z-1000 Pulsed Light system (Xenon Corp, Wilmington, MA, USA), which emits high-intensity polychromatic pulses within a wavelength range of 200–1100 nm, with a pulse width of 360 μs and a pulse frequency of 3 pulses per second. During the experiment, intact tomato fruit samples were placed flat at the center of the experimental platform, oriented perpendicularly to the upper light source with a vertical distance of 10.8 cm. The tomato fruits were irradiated on both sides, meaning that after irradiation on one side was completed, the fruits were flipped and irradiated again with the same dose. Each side was irradiated 12 times, with each irradiation lasting 3.7 s. The total irradiation dose for each single pulse irradiation was 10.32 J/cm^2^. After 1 h of treatment, 3 tomatoes were selected for group G3 for lycopene detection. The remaining four tomatoes were stored in the dark at 4 °C for 15 days before testing, and this part was labeled as group G4. Among the untreated control group tomatoes, three were selected as group G1 for lycopene detection, and the remaining three were designated as group G2 and stored in the dark at 4 °C for 15 days before testing.

### 4.2. Detection of Lycopene in Tomato Samples Using UPLC-MS/MS

The detection of lycopene was performed using ultra-performance liquid chromatography coupled with triple quadrupole mass spectrometry (UPLC-MS/MS) (Sciex, Framingham, MA, USA). Tomato samples stored at −80 °C were first ground into powder using a cryogenic grinder at 50 Hz for 1 min under liquid nitrogen. Subsequently, 200 mg (±5 mg) of the powder was accurately weighed and transferred into a centrifuge tube. Then, 1.5 mL of extraction solvent containing 1% butylated hydroxytoluene (BHT) in a mixture of acetone/hexane/ethanol (1:1:2, *v*/*v*/*v*) was added. The mixture was vortexed to homogenize and then subjected to ultrasonic extraction for 10 min. After that, the sample was centrifuged at 12,000× *g* at 4 °C for 5 min, and the supernatant was collected. The residue was re-extracted twice with 500 μL of acetone/hexane (1:1, *v*/*v*) containing 1% BHT. The combined supernatants were concentrated under reduced pressure using a cold trap. The concentrate was re-dissolved in 100 μL of methanol/tert-butyl methyl ether (3:1, *v*/*v*) containing 1% BHT and filtered through a 0.22 μm organic filter membrane for analysis. Chromatographic separation was achieved on a YMC Carotenoid C30 column (100 × 4.6 mm, 5 μm) (*YMC*, Teknokroma, Barcelona, Spain) with the column temperature maintained at 35 °C. The mobile phase consisted of solvent A (methanol/acetonitrile, 1:1, *v*/*v*) and solvent B (tert-butyl methyl ether). The gradient elution program was as follows: 0–3 min, 100% A; 3–5 min, 100%→30% A; 5–9 min, 30%→5% A; 9–10 min, 5% A; 10–10.1 min, 5%→100% A; 10.1–12 min, 100% A. The flow rate was set at 0.8 mL/min, and the injection volume was 1 μL. Mass spectrometry was performed using an atmospheric pressure chemical ionization (APCI) ion source in positive ion mode. The corona needle current was set at 5 μA, curtain gas at 30 psi, collision gas at 9 psi, and the nebulizer temperature at 550 °C. The analytes were detected in multiple reaction monitoring (MRM) mode.

### 4.3. Transcriptome Analysis

Transcriptome sequencing was performed on 13 samples across four groups, comprising three replicates each for G1, G2, G3, and four for G4. The sequencing was completed at Wuhan Metware Biotechnology Co., Ltd. (Wuhan, China). RNA purity and integrity were assessed using a nanophotometer spectrophotometer and an Agilent 2100 bioanalyzer (Agilent Technologies, Inc., Santa Clara, CA, USA), respectively. Subsequently, the RNA libraries were sequenced on the Illumina Hiseq platform (Illumina Inc., San Diego, CA, USA ). Gene expression levels were quantified using FPKM, with a threshold of |log2Fold Change| ≥ 1 and *p* < 0.05 to identify significantly differentially expressed genes (DEGs). GO and KEGG classifications were used to annotate DEGs.

### 4.4. Protein Extraction, Digestion, and Mass Spectrometry Analysis

Samples were ground in liquid nitrogen and transferred to 5 mL tubes. Protein lysis buffer (8 M urea, containing protease inhibitors) was added, followed by sonication on ice for 2 min and lysis for 30 min. After centrifugation at 12,000× *g* for 30 min at 4 °C, the protein supernatant was collected. Protein concentration was determined using the BCA method with a standard curve of BSA (0–2 mg/mL), measured at 562 nm using a SpectraMax microplate reader (SpectraMax, San Jose, CA, USA). One hundred micrograms of protein were processed with lysis buffer and 100 mM TEAB. TCEP (10 mM) was added and reacted at 37 °C for 60 min, followed by iodoacetamide (40 mM) in the dark at room temperature for 40 min. Samples were precipitated with cold acetone (6:1 *v*/*v*) at −20 °C for 4 h, then centrifuged at 10,000× *g* for 20 min at 4 °C. The pellet was dissolved in 100 mM TEAB and digested with trypsin (1:50 enzyme:protein ratio) at 37 °C overnight. Peptides were dried, resuspended in 0.1% trifluoroacetic acid (TFA), desalted using Oasis hydrophilic lipophilic balance (HLB) (Waters, Milford, MA, USA), and quantified using a peptide quantification kit (Thermo Fisher Scientific, Waltham, MA, USA). Digested peptides were mixed, concentrated by vacuum centrifugation, and resuspended in UPLC loading buffer (2% ACN, 0.1% FA). Peptides were separated on a reverse-phase C18 column (ACQUITY UPLC BEH C18, 1.7 µm, 2.1 mm × 150 mm) (Waters, Milford, MA, USA) using a Vanquish UHPLC system (Thermo Fisher Scientific, Waltham, MA, USA) with a gradient of 2–80% acetonitrile (pH 10) over 47 min at 200 µL/min. Twenty fractions were collected, concentrated, and resuspended in MS loading buffer (0.3% TFA,2% acetonitrile). The fractions were analyzed by MS in DDA mode, capturing the top 20 precursor ions and their MS/MS spectra. The MS1 resolution was 70,000 (*m*/*z* 350–1300) and MS2 resolution was 17,500, using HCD fragmentation.

### 4.5. Metabolite Profiling

Solid samples of 100 mg, added to a 2 mL centrifuged tube and mixed with 800 µL of extraction solution (methanol: water = 4:1 (*v*:*v*)) containing four internal standards (0.02 mg/mL L-2-chlorophenylalanine, etc.) were used for metabolite extraction. Samples were ground by the Wonbio-96c (Shanghai Wanbo Biotechnology Co., Ltd., Shanghai, China) frozen tissue grinder for 6 min (−10 °C, 50 Hz), followed by low-temperature ultrasonic extraction for 30 min (5 °C, 40 kHz). The samples were left at −20 °C for 30 min, centrifuged for 15 min (4 °C, 13,000× *g*), and the supernatant was transferred to the injection vial for LC-MS/MS analysis. The LC-MS/MS analysis of sample was conducted on a UHPLC-Orbitrap Exploris 240 system equipped with an ACQUITY HSS T3 column (100 mm × 2.1 mm i.d., 1.8 μm; Waters, USA). The mobile phases consisted of 0.1% formic acid in water:acetonitrile (2:98, *v*/*v*) (solvent A) and 0.1% formic acid in acetonitrile (solvent B). The flow rate was 0.40 mL/min and the column temperature was 40 °C. The injection volume was 5 μL. The R package “ropls” (Version 1.6.2) was used to perform PLS-DA, and a 7-cycle interactive validation was used to evaluate the model’s stability. Significantly different metabolites were identified based on a variable importance in projection VIP > 1.0 from the PLS-DA model and a *p*-value < 0.05 from Student’s *t*-test. Differential metabolites between the two groups were mapped to their biochemical pathways using metabolic enrichment and KEGG pathway analysis.

### 4.6. Integration of Transcriptome, Proteome, and Metabolome Data

Statistical integration analysis of transcriptomic, proteomic, and metabolomic data was performed as follows: a consolidated list of DEGs, DEPs, and DEMs in the G3 and G4 groups was prepared. The Spearman correlation algorithm (rcorr R package, v5.2.3) was used to calculate correlation coefficients(r) and *p*-values for each gene, protein, and metabolite to ensure dependency or mutual influence among these variables. Statistically integrated genes, proteins, and metabolites (*p* < 0.001 and |r| > 0.95) were selected for further functional analysis. The correlation network was visualized using Cytoscape (version 3.9.1). KEGG pathway enrichment analysis was performed separately for DEGs, DEPs, and DEMs. Pathways shared by DEGs and DEPs, DEGs and DEMs, or DEPs and DEMs were selected for analysis of their status in the G3 and G4 groups. The detailed workflow parameters, including statistical thresholds and normalization methods, are provided in Supplementary Table S2.

### 4.7. qRT-PCR

To validate the reliability of our transcriptomic data and to specifically investigate the key lycopene biosynthesis pathway, we selected eight crucial genes for qRT-PCR analysis. These genes include *Solyc03g031860.3*, *Solyc02g069460*.3, *Solyc12g099260*.2, *Solyc01g097810*.3, *Solyc05g053300*.3, *Solyc04g050930*.3, *Solyc08g081570*.3, and *Solyc03g115980*.1. Total RNA was extracted from the material using the RNAprep Pure Plant Total RNA Extraction Kit (Tiangen, Beijing, China). The reverse transcription of single-stranded DNA was synthesized using the HiScript III RT SuperMix for qPCR (+gDNA wiper) (Vazyme, Nanjing, China), following the manufacturer’s protocol. Quantitative reverse transcription-polymerase chain reaction (qRT-PCR) was conducted with three biological replicates. qRT-PCR was performed with three biological replicates using a Roche LightCycler 480 real-time PCR instrument (Roche Diagnostics GmbH, Mannheim, Germany). Relative quantitative analysis of gene expression was performed using the 2−ΔΔCT method to analyze the expression levels of genes involved in lycopene biosynthesis. Primers were designed using multiPrime [33] and the sequences of the primers are shown in Supplementary Table S3.

## 5. Conclusions

This study systematically elucidated the molecular mechanisms underlying lycopene synthesis regulated by pulsed light treatment, integrating high-throughput data from transcriptomics, proteomics, and metabolomics. Multi-omics analyses indicated that gene expression, protein accumulation, and metabolite levels in the carotenoid biosynthesis pathway were coordinately upregulated in tomatoes after pulsed light treatment, with significant activation particularly in the TCA cycle, terpenoid backbone biosynthesis, and photosynthesis-antenna protein-related pathways. The research findings highlighted that the reprogramming of energy metabolism (including oxidative phosphorylation and carbon fixation pathways) and the synthesis of secondary metabolites (such as porphyrin metabolism and glutathione metabolism) played crucial roles in lycopene accumulation induced by pulsed light. Further integration of multi-omics analyses revealed that pulsed light treatment significantly enhanced lycopene content in tomatoes during postharvest storage by synchronously regulating the activity of the photosynthetic system, carbohydrate metabolism, and terpenoid synthesis network. This study not only provides a theoretical basis for quality regulation during tomato postharvest storage but also offers significant scientific reference value for the development of new tomato preservation technologies based on pulsed light treatment.

## Data Availability

The original data presented in this study are openly available in public repositories. The proteomics data can be accessed through ProteomeXchange with identifier PXD070355. The RNA-seq data have been deposited in the NCBI Sequence Read Archive (SRA) under project accession PRJNA1354624. The metabolomics data are available in the MetaboLights database under accession number MTBLS13258.

## Supplementary Materials

The following supporting information can be downloaded at: https://www.mdpi.com/article/10.3390/ijms262411828/s1.
